# JARID2, a novel regulatory factor, promotes cell proliferation, migration, and invasion in oral squamous cell carcinoma

**DOI:** 10.1186/s12885-024-12457-6

**Published:** 2024-07-03

**Authors:** Yuxi Cheng, Zhengzheng Song, Jingyi Cheng, Zhangui Tang

**Affiliations:** 1 Xiangya Stomatological Hospital & Xiangya School of Stomatology, Central South University, Changsha, Hunan 410008 China; 2 Hunan Key Laboratory of Oral Health Research & Hunan Clinical Research Center of Oral Major Diseases and Oral Health & Academician Workstation for Oral-maxilofacial and Regenerative Medicine, Changsha, Hunan 410008 China

**Keywords:** JARID2, Oral squamous cell carcinoma, Tumor progression, Biological behavior, Immune infiltration, Tumor microenvironment

## Abstract

**Background:**

Accurate regulation of gene expression is crucial for normal development and function of cells. The prognostic significance and potential carcinogenic mechanisms of the related gene JARID2 in OSCC are not yet clear, but existing research has indicated a significant association between the two.

**Methods and materials:**

The relationship between the expression of the JARID2 gene in tumor samples of OSCC patients and clinical pathological factors was analyzed using immunohistochemistry experiments and RT-qPCR analysis. Based on the clinical pathological data of patients, bioinformatics analysis was conducted using public databases to determine the function of JARID2 in OSCC. Knockdown OSCC cell lines were constructed, and the impact of JARID2 on the biological behavior of OSCC cell lines was assessed through CCK-8, wound healing assay, and transwell analysis.

**Results:**

Immunohistochemistry experiments confirmed the correlation between JARID2 and the prognosis of OSCC patients, while RT-qPCR experiments demonstrated its expression levels in tissue and cells. CKK-8 experiments, wound healing assays, and Transwell experiments indicated that knocking down JARID2 had a negative impact on the proliferation, invasion, and migration of OSCC cells. Bioinformatics analysis results showed that the expression of JARID2 in OSCC is closely associated with patient gene co-expression, gene function enrichment, immune infiltration, and drug sensitivity.

**Conclusion:**

Our study indicates that JARID2 is a novel prognostic biomarker and potential therapeutic target for OSCC.

## Introduction

Oral squamous cell carcinoma (OSCC) is a prevalent form of cancer originating from the mucosal epithelium of the oral cavity. It has a high global incidence, with an estimated 377,713 new cases reported annually [12]. Unfortunately, OSCC is often diagnosed at advanced stages, which limits treatment options and leads to a grim prognosis. Surgical resection is typically the primary treatment for OSCC, followed by adjuvant radiotherapy or a combination of chemotherapy and radiotherapy, depending on the disease stage. Despite significant advancements in understanding the mechanisms involved in OSCC, the five-year survival rate remains approximately 50%. Therefore, it is crucial to comprehend the underlying biological processes driving OSCC in order to develop effective treatments for this condition [4, 8].

Accurate regulation of gene expression is crucial for proper cellular development and functionality. Gene regulation occurs at various levels, including DNA-binding transcription factors, chromatin structure, and post-translational modifications of histones [2]. The Polycomb repressive complexes (PRCs) play a vital role in regulating gene expression at the chromatin level and are indispensable during development. In mammals, PRCs have the ability to selectively target specific sites in the genome and, through feedback mechanisms, establish Polycomb chromatin domains that control transcription. Polycomb proteins typically assemble into two protein complexes, namely Polycomb Repressive Complex 1 (PRC1) and PRC2. PRC1 and PRC2 carry out post-translational modifications on histones. PRC1 monoubiquitinates histone H2A at Lys119 (H2AK119ub1), while PRC2 trimethylates histone H3 at Lys27 (H3K27me3) and also mono- and dimethylates it. Furthermore, PRC1 and PRC2 tend to cluster in close proximity at the same genomic loci, forming Polycomb chromatin domains that exert transcriptional repression effects [1, 3].

JARID2 is an interacting component of PRC2 that mediates the H3K27me3, leading to the downregulation of PTEN. Multiple studies conducted in mice and humans have revealed the significance of the N-terminal region of JARID2 in PRC2 recruitment and regulation of PRC2 activity. This N-terminal region encompasses a nucleosome-binding domain and an RNA-binding domain, which collectively influence the interaction between PRC2 and genomic DNA. JARID2 has been identified as a substoichiometric component of PRC2 and contributes to the targeted functionality of PRC2, closely associated with various physiological processes in cells. In cardiac cells, JARID2 has been demonstrated to govern the cell cycle by regulating cyclin D1, whereas in skeletal muscle cells, it modulates the cell cycle in a PRC2-dependent manner. PRC2/JARID2 is also a recent hotspot in oncology research [5, 9, 11, 14]. However, the prognostic significance and potential carcinogenic mechanisms of JARID2 in OSCC are not yet clear. We hope that by exploring the biological function of this gene, we can provide new insights for future OSCC treatment and diagnosis, and improve patient prognosis.

In our study, we observed upregulation of JARID2 expression in OSCC cells. The elevated expression of JARID2 was identified as an independent prognostic factor, indicating a poorer overall survival rate in OSCC patients. Overexpression of JARID2 promoted the viability, invasion, and migration of OSCC cells, and it was closely associated with tumor immune microenvironment and drug sensitivity.

## Materials and methods

### Ethical Statement

The normal oral mucosa and OSCC specimens utilized in this study were provided by the Oral and Maxillofacial Surgery Department at Xiangya Stomatological Hospital, Central South University. The diagnosis of OSCC samples was performed by experienced clinicians and pathologists, adhering to the diagnostic criteria outlined in the 2017 American Cancer Joint Commission and the 2018 National Comprehensive Cancer Network guidelines. Ethical clearance for the study was obtained from the Medical Ethics Committee of Xiangya Stomatological Hospital, Central South University (Approval No: 20,230,078). Written informed consent was obtained from all participating patients.

### Patients and specimens

A total of 55 OSCC samples were collected from patients who underwent surgery at the Xiangya Stomatological Hospital of Central South University from January 2013 to December 2019. Patients meeting the following criteria were included:

1. All patients had an initial pathological diagnosis of OSCC.
2. All patients had not received chemotherapy, immunotherapy, or radiation therapy.
3. All patients had no contraindications and underwent surgical treatment.

All patients in this study underwent extensive tumor resection. Patients with potential cervical lymph node metastasis underwent modified radical neck dissection, while T2-3N0 patients underwent selective lymph node dissection (Levels I-III, V). T1N0 patients were monitored and followed up. Clinical staging was determined based on the AJCC TNM staging system using MRI scans. All patients were followed up every 2 months in the first year after surgery, every 3 months in the following 2–3 years, and every 6 months in the subsequent 4–5 years, until the patient’s death or January 2023. After an 18-month follow-up period, T1N0 patients without cervical lymph node metastasis were considered lymph node negative. Overall survival was defined as the time from the first surgery to the date of death.

### Data acquisition

The OSCC patient data was acquired from the HNSC dataset within the Cancer Genome Atlas (TCGA) database, accessible via https://portal.gdc.cancer.gov/projects/TCGA-HNSC. The dataset encompassed RNA transcriptome sequencing data, relevant clinical information, and mutation data. Samples originating from primary sites outside of the oral cavity (such as the hypopharynx, larynx, oropharynx, and tonsil) were excluded from the analysis. A total of 341 OSCC patient RNA expression data, along with their corresponding clinical information, were included, as well as 331 OSCC patient mutation data, for subsequent analysis.

### Differential expression and co-expression analysis

Initially, we conducted an analysis of JARID2 differential expression in normal and OSCC samples using the “limma” R package, and visualized the results through a box plot. Subsequently, co-expression analysis of the JARID2 gene was conducted utilizing RNA-seq data from OSCC patients, with a filter condition set at a p-value < 0.05 threshold. For correlation analysis of JARID2, we employed R packages “corrplot” and “circlize” to generate a heatmap and circular plot, respectively.

### Functional enrichment analysis

Based on the expression level of JARID2, the patients were categorized into high and low expression groups. To assess the differences in signaling pathways between these two groups, we obtained the annotated gene set from the Molecular Signatures Database (MsigDB). Utilizing GSEA (Gene Set Enrichment Analysis), we conducted an analysis to identify pathway gene sets that exhibited significant enrichment (adjusted p-value < 0.05). These gene sets were subsequently ranked based on consistency scores. Additionally, we employed the GSVA (Gene Set Variation Analysis) algorithm to comprehensively score each gene set, offering a comprehensive evaluation of potential biological functional changes between the high and low expression groups. These findings were visualized through bar charts.

### ssGSEA immune infiltration analysis

We utilized the ssGSEA (single-sample Gene Set Enrichment Analysis) algorithm to analyze the RNA-seq data from OSCC patients. This allowed us to quantify immune cell infiltration by leveraging a gene set consisting of immune cell markers, thereby inferring the relative proportions of 28 immune cell types in OSCC samples. To examine the relationship between JARID2 expression levels and immune cell content, we performed correlation analysis using the Spearman rank correlation test.

### Mutation and drug sensitivity analysis

To illustrate the mutation landscape and compare the disparities in mutated genes between the high and low expression groups, we employed the “ComplexHeatmap” R package. Additionally, leveraging the Genomics of Drug Sensitivity in Cancer (GDSC) database, which is the largest pharmacogenomics database available at https://www.cancerrxgene.org/, we predicted the chemotherapy sensitivity for each OSCC patient using the “pRRopetic” R package. The IC50 value for each specific chemotherapy drug was estimated using regression methodology, and to evaluate the regression and prediction accuracy, we conducted ten cross-validation tests on the GDSC training set.

### Cell culture and transfection

The HACAT human cell line and OSCC cell lines, namely SCC25 and HN30, were provided by the China Center for Type Culture Collection. These cell lines were cultured in Dulbecco’s modified Eagle’s medium (DMEM; Biological Industries, Israel; C3120-0500), supplemented with 10% fetal bovine serum (Biological Industries, Israel; 04-001-1 C) and 1% penicillin-streptomycin (Biological Industries, Spain, 100 units/ml). The cells were maintained at 37 °C, 5% CO2, and 95% relative humidity. Routine medium replacement was conducted every 48 h, and cell passages were performed when reaching 80% confluence.

For the SCC25 and HN30 cell lines, the following categorizations were applied: blank (non-transfected), ncJARID2 (transfected with plasmid or siRNA containing empty vector), Pex-3 (transfected with overexpressed JARID2 plasmid), and si-JARID2 (transfected with JARID2 siRNA). Lipofectamine™ 3000 (Invitrogen, USA, L3000015) was used for transfection, following the manufacturer’s instructions. The overexpression plasmid for JARID2 and JARID2 siRNA were obtained from Suzhou GenePharma Co. (GenePharma, Jiangsu, China, B8238). Transfected cells were collected at 48 and 72 h post-transfection for quantitative real-time polymerase chain reaction (RT-qPCR) and other experimental assessments.

### RNA extraction and RT-qPCR analysis

Total RNA was extracted from the samples, and then converted into complementary DNA (cDNA) using TRIzol reagent (Invitrogen, USA, 50,175,111) following the manufacturer’s instructions (Vazyme, China, R123-01). To measure the mRNA levels, a quantitative real-time polymerase chain reaction was conducted using the SYBR Green quantitative PCR kit (Vazyme, China, Q711-02/03). GAPDH was used as the internal reference. The primer sequences used in this study can be found in the Supplementary File (Sangon Biotech, China).

### Cell proliferation assay

The cells were seeded at a density of 1 × 10^4^/well in 96-well plates. Cell viability was evaluated by measuring absorbance using a Cell Counting Kit-8 (Dojindo, Japan, CK04). Transfected cells were incubated for 2 h at 37 °C at four different time points (0, 24, 48, and 72 h). An enzyme marker and microplate reader (BioTek, USA) were utilized to measure the optical density (OD) at 450 nm. The experiment was conducted three times, and the average value was used for data analysis.

### Wound healing test

The wound healing assay is a commonly used method for assessing cell migration ability in vitro. Transfected cells were cultured on plates until they reached 90% confluency. To create a “wound” in the cell layer, a pipette tip was used to scrape the cells along a parallel line at the base of the culture dish. The progress of wound closure was monitored using microscopy, and images were captured at different time intervals. To quantify cell migration into the wound area, cell counting was periodically performed at specific locations along the line to estimate the rate of wound healing. This assay was repeated three times, and the average measurements were calculated.

### Transwell assay

For this assay, a transwell (Corning, USA, 3422) with a Matrigel-coated membrane (BD, USA, 356,234) was utilized to establish two chambers: the upper and lower chambers. Cells were placed in the upper chamber, while the lower chamber contained a serum-containing medium. The cells were allowed to invade and migrate through the Matrigel membrane. Following an incubation period of 48–72 h, the cells on the underside of the membrane were fixed, stained with crystal violet, and examined under a microscope. The experiment was repeated three times to observe and quantify the number of cells invading and migrating through the membrane. The average count across five different fields of view was then calculated.

### Immunohistochemistry

A total of 35 pairs of OSCC and normal mucosa samples were subjected to immunohistochemical staining using a universal two-step detection kit (ZSGB-BIO, China, PV-9000). The tissue samples were fixed in 10% formaldehyde, embedded in paraffin, and then sectioned consecutively into thin sections of approximately 3 μm. These sections were baked at 65 °C for 2 h, dewaxed, hydrated, and underwent heat treatment at 100 °C in a citrate buffer solution (pH = 6.0) for 15 min. After incubating with 3% hydrogen peroxide for 10 min, the sections were then incubated overnight at 4 °C with an antibody against JARID2 (GeneTex, USA, GTX129019, 1:500). Subsequently, the sections were incubated with a reaction enhancement solution and an enzyme-labeled polymer of goat anti-mouse/rabbit immunoglobulin G following washing steps. Protein expression was evaluated by comparing the staining intensity between the OSCC and normal mucosa samples.

Two experienced pathologists (Zhigang Yao and Long Li) independently evaluated the results of immunohistochemical staining and achieved final consensus for each scoring under discussion using a microscope. The staining intensity and the percentage of stained cells were evaluated using the Image-Pro Plus 6.0 software. Staining intensity was scored as follows: 0 (no staining), 1 (weak staining), 2 (moderate staining), and 3 (strong staining). The percentage of stained cells was scored on a scale of 0 to 100, corresponding to a score of 0 to 100. All images were analyzed using the same settings. The final immunoreactivity score, representing the integrated index of the percentage of positive cells and staining intensity, ranged from 0 to 300. JARID2 expression levels were categorized as follows: negative (-, score 0–5), low expression (+, score 6-100), medium expression (++, score 101–200), and high expression (+++, score 201–300).

### Statistical analyses

The immunohistochemical results were statistically analyzed using statistical software SPSS 22.0 (IBM). The Fisher exact chi-square test was employed to assess the differences in JARID2 protein expression among different clinical pathological classifications. Prognostic factors were analyzed using Cox regression models with the “Forward: LR” method. The Kaplan-Meier method was used to evaluate the overall survival rate of patients with different JARID2 expressions (According to the IHC score), and the Tarone test was employed for comparison. The paired sample t-test was used to compare the means between the two groups. Results were considered statistically significant at a two-sided p-value < 0.05. GraphPad Prism 9.0 was used for data analysis in other biological experiments. Analysis of variance (ANOVA) was performed to evaluate the data, and mean ± SD was used for presentation. Differences were considered statistically significant at *p* < 0.05. Six photographic fields were captured from all the images and used for analysis. Each experiment was repeated three times. The paired sample t-test was used to compare the means between the two groups. Results were considered statistically significant at a two-sided p-value < 0.05. The bioinformatics analyses were performed using R software (version 4.1.0). The Wilcoxon rank-sum test was used to analyze non-normally distributed variables, and the Kaplan-Meier technique was used to generate survival curves. The log-rank test was used to analyze differences. Correlation coefficients were calculated using Spearman methods.

## Results

### JARID2 is overexpressed in OSCC samples and is associated with a poor prognosis

**Fig. 1:**
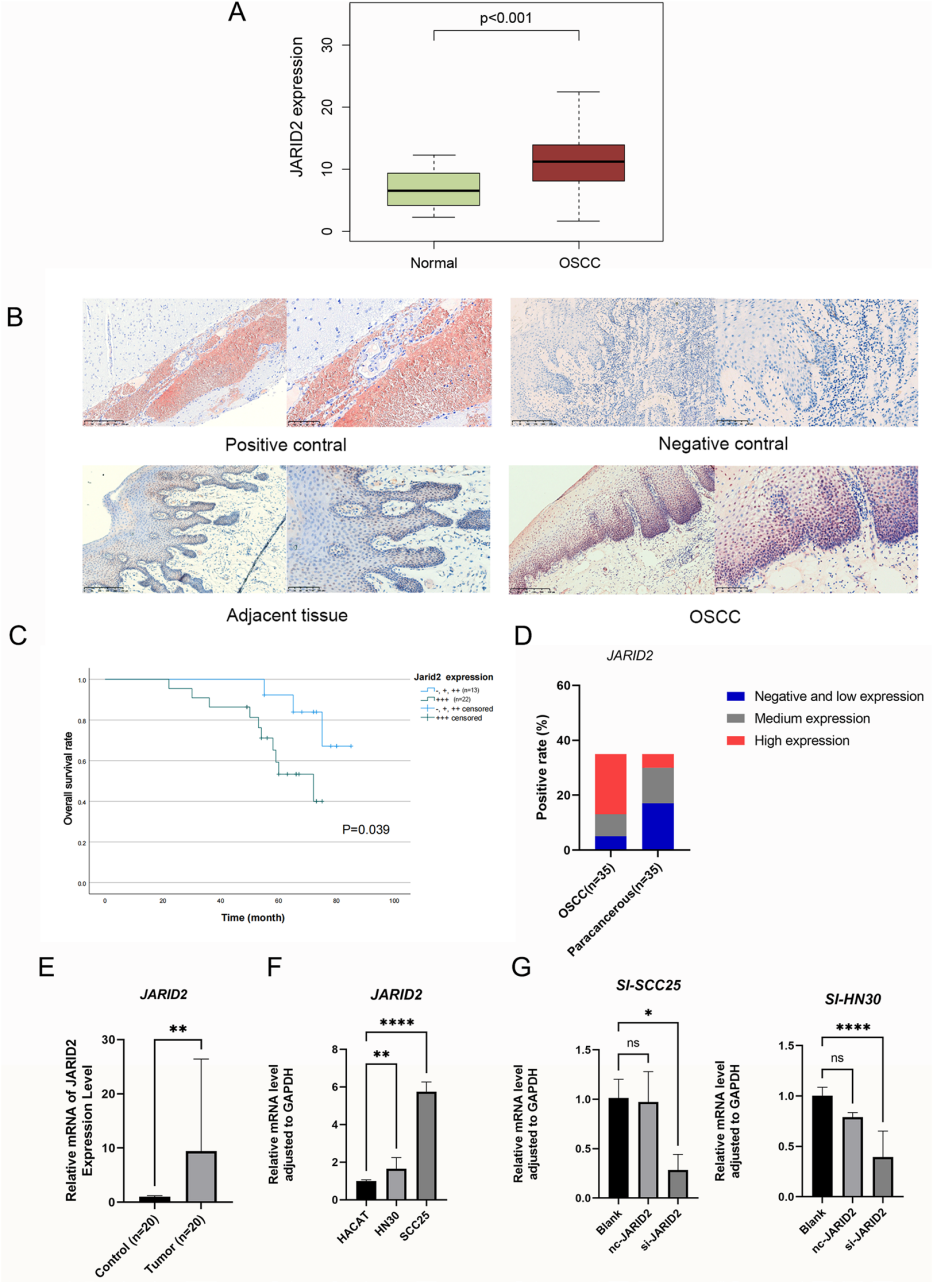
(**A**) TCGA transcriptome data showed that JARID2 had a significantly higher expression in OSCC samples than in normal samples. (**B**) Immunohistochemical staining showed that JARID2 was expressed in adjacent normal oral mucosa (*n* = 35) and OSCC samples (*n* = 35); Using human brain tissue as a positive control. Use non immune IgG instead of primary antibody as negative control (left: 100 x, right: 200 x). (**C**) Overall survival in patients with OSCC according to JARID2 protein expression. (**D**) JARID2 expression was higher in normal tissue adjacent to cancer in patients than in cancer tissue. (**E**) RT-qPCR showed that the expression of JARID2 was significantly lower in adjacent normal oral mucosa (*n* = 20) than in OSCC samples (*n* = 20). (**F**) Expression of JARID2 in HACAT, SCC25, and HN30 cells. (**G**) Evaluation of knockout and overexpression efficiency by RT-qPCR. * *p* < 0.05, **** *p* < 0.0001

Initially, we examined data from the TCGA database to establish that the expression of JARID2 was elevated in tumor samples compared to normal samples or adjacent normal samples from patients with OSCC (Fig. 1A). Immunohistochemical staining of 35 OSCC samples and 35 normal oral mucosal specimens provided further confirmation that JARID2 expression was significantly higher in OSCC than in normal oral mucosal specimens (Fig. 1B). We analyzed the correlation between JARID2 protein expression and clinical pathological characteristics of OSCC patients. The results showed that the expression of JARID2 was related to age (*p* < 0.001), pathological grade (*p* = 0.441), T stage (*p* = 0.006), and lymph node metastasis factors (*p* = 0.029) (Table 1). Kaplan-Meier survival plot also showed that OSCC patients with high expression of JARID2 protein had a significantly worse prognosis (*p* = 0.039) (Fig. 1C), and JARID2 expression was higher in normal tissue adjacent to cancer in patients than in cancer tissue (Fig. 1D).Differential gene expression analysis based on the TCGA database revealed a significant increase in JARID2 expression in OSCC samples compared to normal oral mucosal samples (*p* < 0.001) (Fig. 1E). RT-qPCR analysis demonstrated a notably higher expression level of JARID2 in SCC25 and HN30 cells compared to HACAT cells (*p* < 0.0001) (Fig. 1F).

**Table 1:** The relationship between JARID2 expression and clinicopathological factors in patients with OSCC

		JARID2 expression level				
**Factors**	**Number**	**Negative (-) and low expression (+)**	**Medium expression (++)**	**High expression (+++)**	**Fisher’s exact** x**²**	**P value**
Total	35	5	8	22		
Age (years)					25.457	<0.001
<50	16	5	8	3		
≥50	19	0	0	19		
T stage					9.476	0.006
T1/T2	15	4	6	5		
T3/T4	20	1	2	17		
Lymph node metastasis					6.714	0.029
N0	12	3	5	4		
N+	23	2	3	18		
Distant metastasis					1.197	1.000
M0	34	5	8	21		
M1	1	0	0	1		
Pathology grade					1.617	0.441
I	31	5	8	18		
II	4	0	0	4		
Neurovascular invasion					0.430	1.000
-	29	4	7	18		
+	6	1	1	4		

### Co-expression analysis

**Fig. 2:**
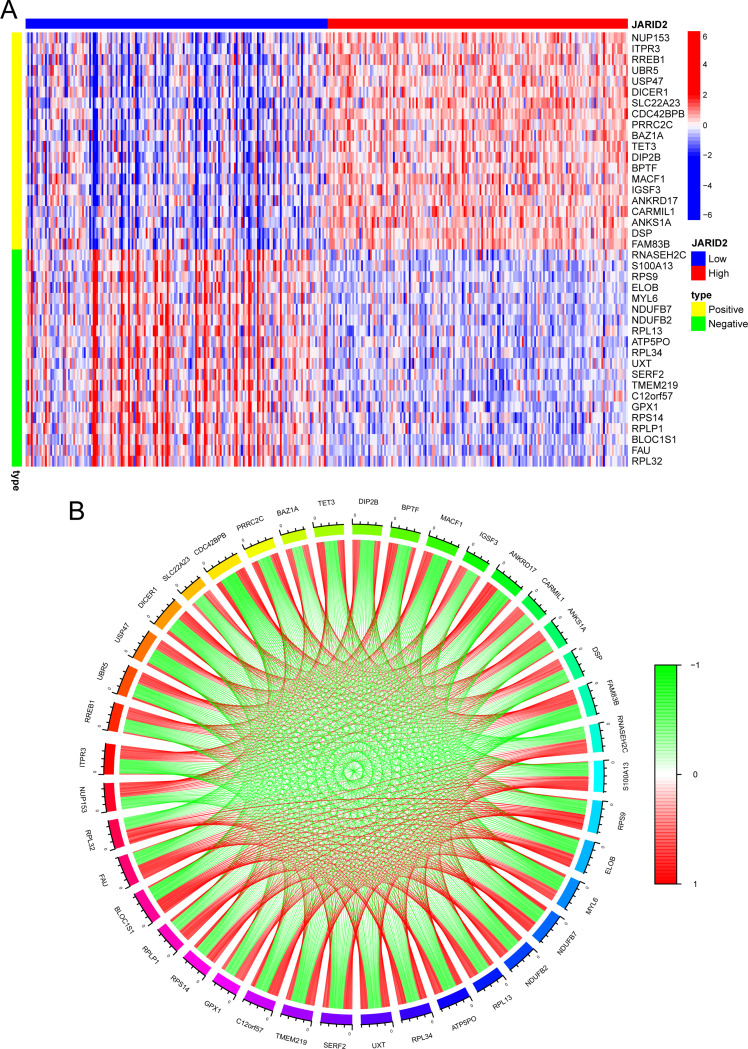
(**A**) Heatmap of the top 20 co-expressed genes of JARID2. (**B**) The co-expression network of JARID2

To find potential gene interactions, we identified genes co-expressed with JARID2 and described a co-expressed gene network. The top 20 genes with the largest absolute positive and negative correlation coefficients were displayed (Fig. 2A). The expressions of NUP153, ITPR3, RREB1, UBR5, USP47, etc., were significantly positively correlated with the expression of JARID2, indicating a functional synergy, while the expressions of RNASEH2C, S100A13, RPS9, ELOB, MYL6, etc., were significantly negatively correlated with the expression of JARID2, indicating a functional antagonism. The co-expression network of JARID2 was exhibited by a cyclic graph (Fig. 2B).

### Functional enrichment analysis

**Fig. 3:**
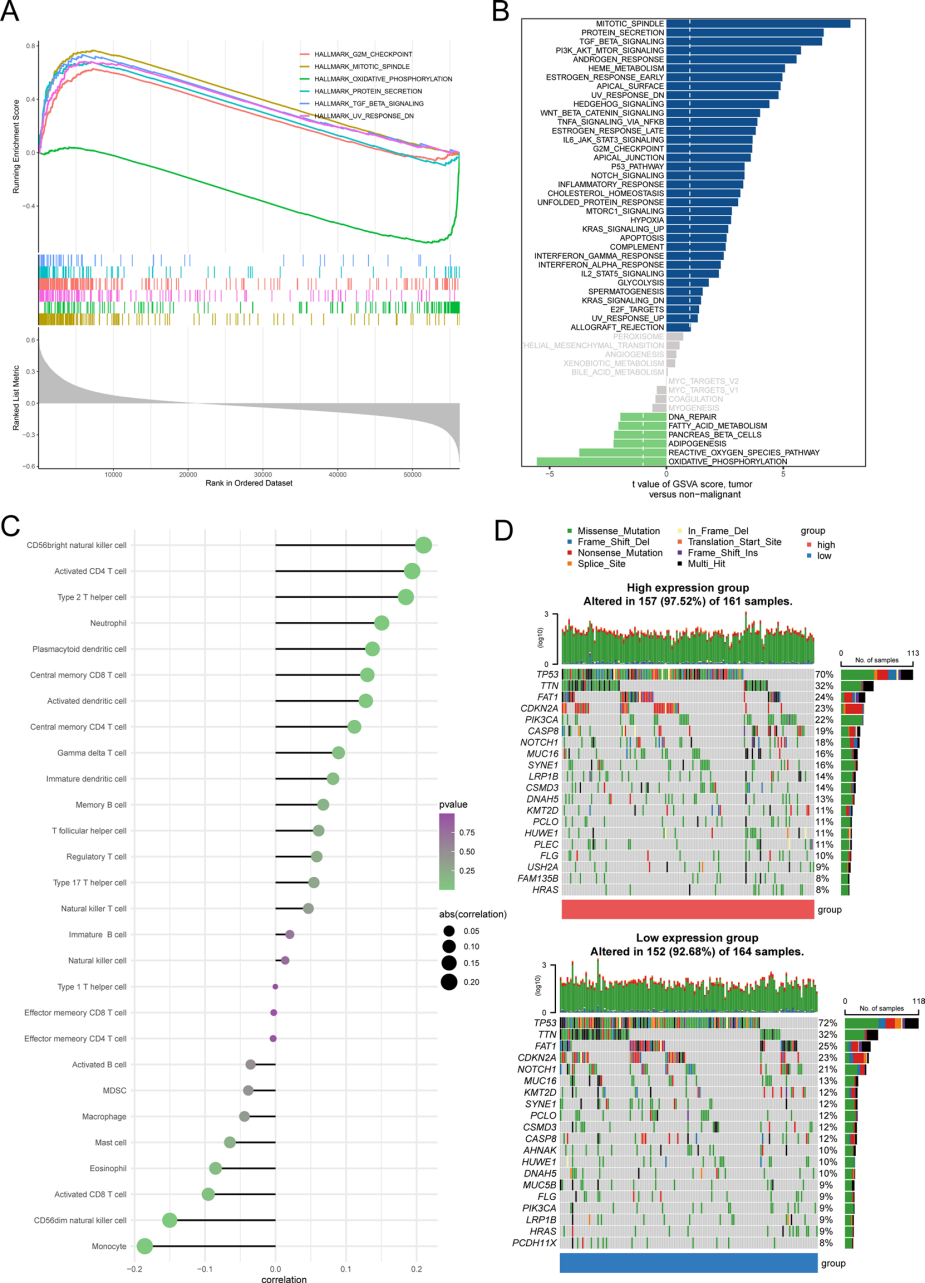
(**A**) Functional enrichment analysis based on GSEA between high and low JARID2 expression groups. (**B**) Functional enrichment analysis based on GSVA between high and low JARID2 expression groups. (**C**) The correlation of JARID2 expression with 28 immune cell types. (**D**) Waterfall plots showing the differences in gene mutation between high and low expression groups

We performed GSVA & GSEA analysis to clarify the possible pathways and biological processes involved with JARID2. We showed the top 6 most significantly enriched pathways based on GSEA results, which were MITOTIC_SPINDLE, OXIDATIVE_PHOSPHORYLATION (negatively correlated), UV_RESPONSE_DN, G2M_CHECKPOINT, PROTEIN_SECRETION, and TGF_BETA_SIGNALING, respectively (Fig. 3A). The result of GSVA was consistent generally, with MITOTIC_SPINDLE, PROTEIN_SECRETION, and TGF_BETA_SIGNALING as the top 3 pathways enriched in high JARID2 expression group and OXIDATIVE_PHOSPHORYLATION as the top 1 pathway enriched in low expression group (Fig. 3B).

### Immune infiltration analysis

We analyzed the correlation between immune cell infiltration and JARID2 expression in OSCC samples, and the results suggested that different level of JARID2 expression could be associated with distinct immune landscape. We presented the results as a bubble chart (Fig. 3C). A number of immune cells, such as CD56bright natural killer cell, activated CD4 T cell, type 2 T helper cell, neutrophil, plasmacytoid dendritic cell, central memory CD8 T cell, activated dendritic cell, and central memory CD4 T cell, had a significant positive correlation with JARID2 expression. However, immune cells such as monocyte and CD56dim natural killer cell were significantly negatively correlated with JARID2 expression.

### Mutation analysis

We presented waterfall plots showing the different gene mutation landscapes of high and low JARID2 expression groups (Fig. 3D). The results reflected a higher general gene mutation frequency in high JARID2 expression group (97.52%) than that in low JARID2 expression group (92.68%). Diverse types of genes, such as PIK3CA, CASP8, SYNE1, and LRP1B, mutated more frequently in high expression group (22%, 19%, 16%, 14%, compared to 9%, 12%, 12%, 9% in low expression group, respectively).

### Drug sensitivity analysis

**Fig. 4:**
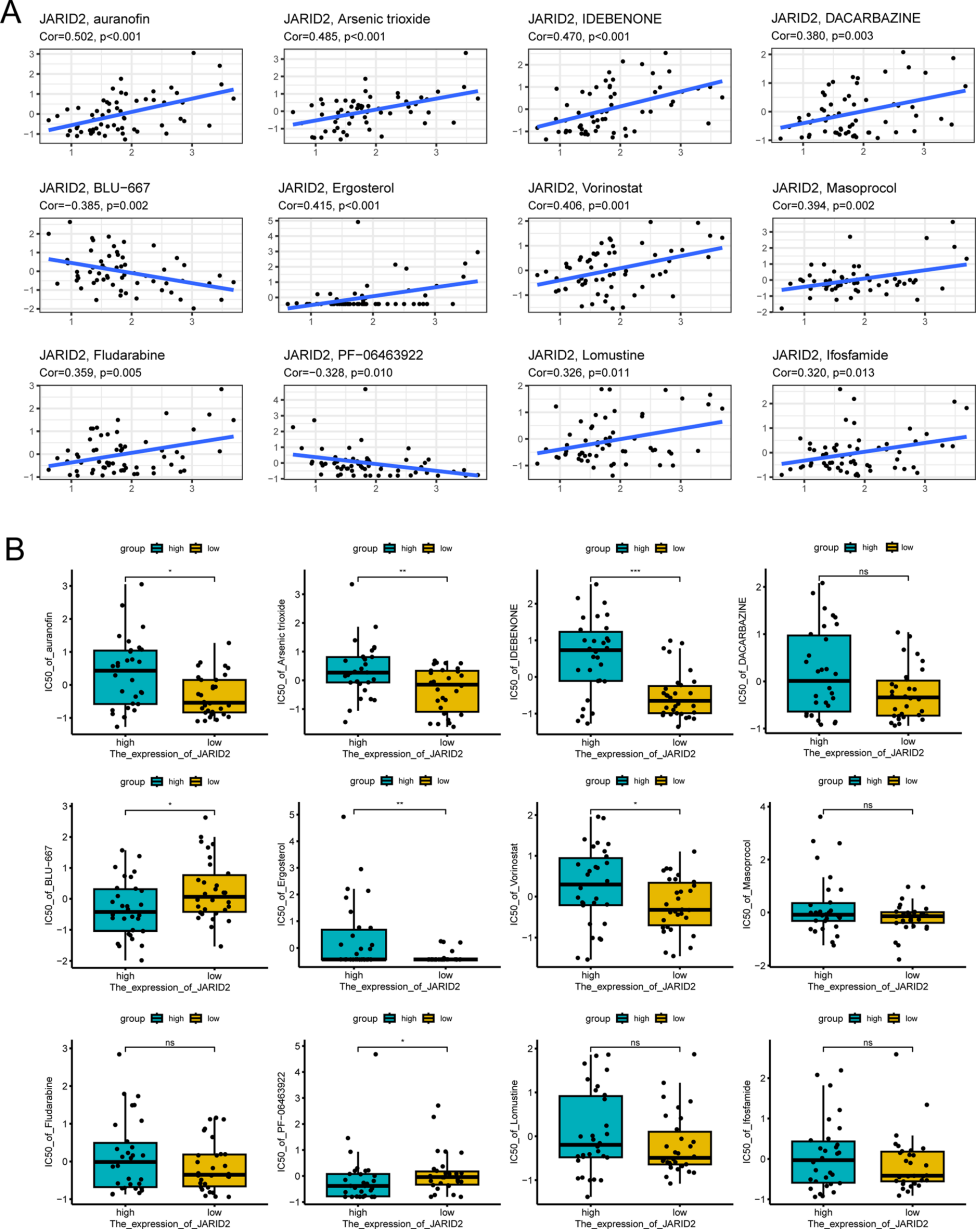
(**A**) The correlation between drug sensitivity and JARID2 expression. (**B**) The difference in drug sensitivity between high and low JARID2 expression group

The correlation between drug sensitivity and JARID2 expression was analyzed based on GDSC database to clarify whether it is possible to predict the drug responses by JARID2 expression. The results showed that the sensitivities of various drugs were significantly correlated with the expression level of JARID2, and we presented the top 12 drugs (Fig. 4A). However, among the 12 drugs, only 7 drugs (auranofin, arsenic trioxide, idebenone, BLU-667, ergosterol, vorinostat, and PF-06463922) showed significant differences in IC50 between high and low JARID2 groups (Fig. 4B), which could be considered as the drugs that applied according to JARID2 expression levels.

### JARID2 affects the proliferation, invasion, and migration of OSCC cells

**Fig. 5:**
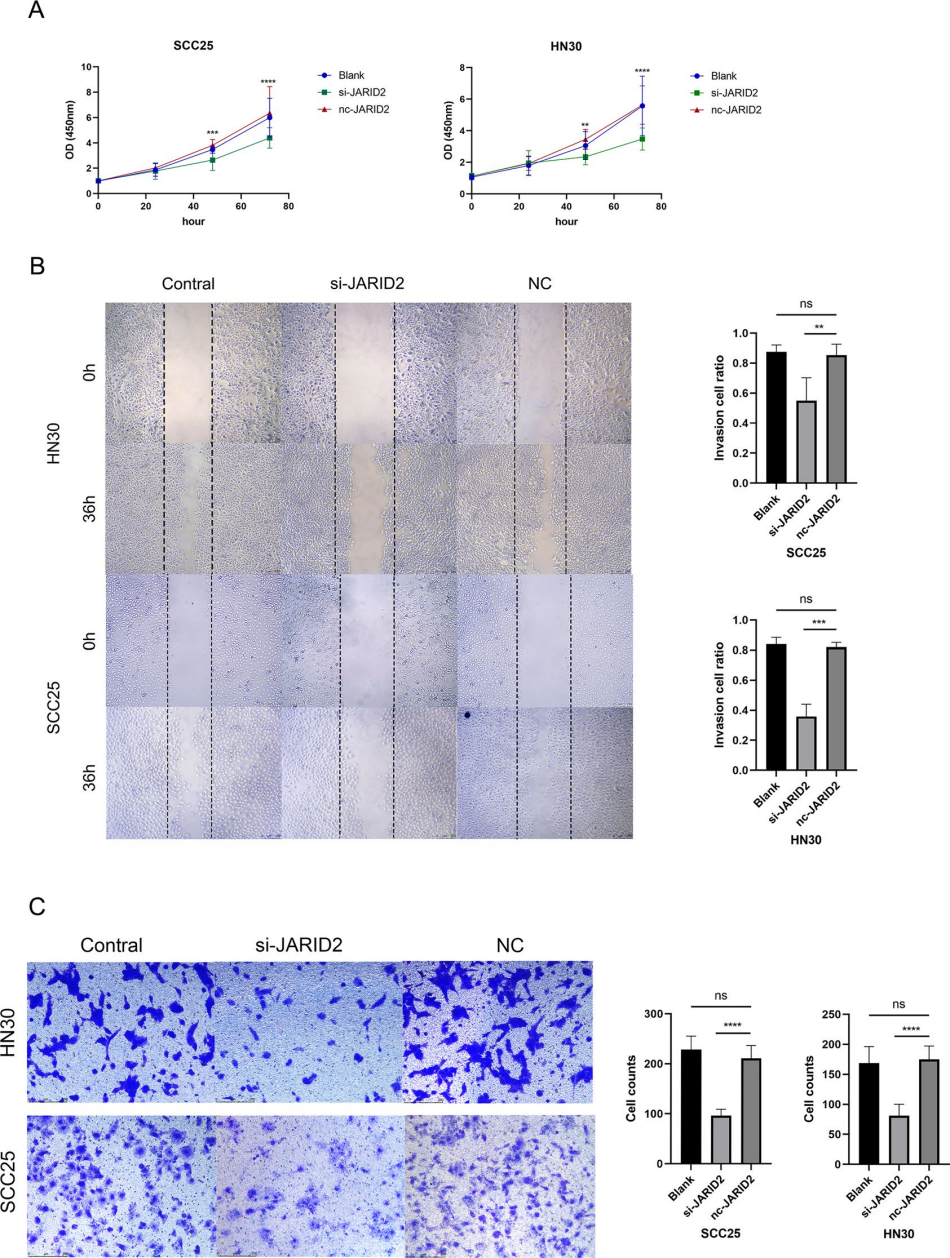
(**A**) The CCK-8 method was used to detect the proliferation of JARID2 knockdown SCC25 and HN30 cells compared to the nc group. (**B**) Migration ability of JARID2 knockdown SCC25 and HN30 cells compared to the nc group by wound healing test. (**C**) Transwell assays were performed to indicate the invasion of JARID2 knockdown SCC25 and HN30 cells compared to the nc group. ns: not significant; ** *p* < 0.01, *** *p* < 0.001, and **** *p* < 0.0001

JARID2 expression in SCC25 and HN30 cells was modulated using small interfering RNA (siRNA) targeting JARID2 to achieve knockdown. The effectiveness of these manipulations was confirmed through RT-qPCR analysis (Fig. 1G). In the cell proliferation experiment utilizing the CCK-8 assay, it was observed that JARID2 knockdown significantly inhibited the proliferation of SCC25 and HN30 cells (Fig. 5A). Moreover, the wound healing and transwell assays (Fig. 5B-C) demonstrated a notable decrease in the invasion and migration abilities of HN30 and SCC25 cells in the si-JARID2 group compared to the nc group (*p* < 0.05).

## Discussion

Mammalian PRCs consist of catalytic cores that, when combined with auxiliary proteins, form distinct PRC1 and PRC2 complexes. Different PRCs exhibit unique compositions, structures, and activities, forming the basis for gene regulation in mammals. PRC1 possesses E3 ubiquitin ligase activity, allowing it to monoubiquitinate histone H2A. The recognition specificity of PRC1 for Lys119 on H2A arises from the interaction between PRC1 and the nucleosome-binding surface. The catalytic core of PRC1 is composed of RING1 and one of six PCGF (Polycomb group RING finger) proteins. The E2 enzyme interacts with the N-terminal of RING1B and DNA at the nucleosome dimer, directing ubiquitin to H2AK119, ensuring substrate specificity for PRC1 [15, 16].

PRC2 primarily forms a tetrameric core complex, consisting of two distinct lobes: the catalytic lobe and the target regulatory lobe. In the catalytic lobe, EZH2 interacts with nucleosomal DNA and the N-terminal portion of H3 tail, facilitating the proximity of Lys27 to the active site of PRC2 for methylation. The target regulatory lobe includes the N-terminal portion of SUZ12, which interacts with RBBP4 or RBBP7 and various other auxiliary factors, generating PRC2 forms with different biochemical properties [6].

JARID2 plays a crucial role in the binding of PRC2 to its target genes, leading to downregulation of gene expression through H3K27me3. It is highly expressed in various types of cancers, including breast cancer. JARID2 promotes glycolysis, lipid metabolism, proliferation, invasion, and stemness of breast cancer cells. In hepatocellular carcinoma, MiR-22 directly binds to JARID2 and plays an important role in MiR-22-mediated Th17 differentiation regulation [17]. Additionally, JARID2 may serve as a downstream target gene in patients with cleft palate. At present, it remains unclear whether JARID2 can interact with H3K27me3 to regulate the expression of PTEN in OSCC. However, there is existing research in other tumors with similar molecular signaling processes. For example, JARID2 is overexpressed in hepatocellular carcinoma (HCC) tissues [7], where its upregulation promotes EMT by inhibiting the phosphatase and tension homolog (PTEN)-induced AKT hyperactivation, resulting in enhanced invasion of HCC cells. Knockdown of JARID2 inhibits transforming growth factor-beta (TGF-β)-induced EMT in colon cancer HT29 cells and lung cancer A549 cells [10, 13]. Knockdown of JARID2 has been reported to impair the invasive and tumorigenic abilities of bladder cancer cells, reducing the number of tumor-initiating cells, suggesting an important role of JARID2 in the progression of OSCC [18]. Therefore, we explore the function of JARID2 in OSCC and further investigate the potential mechanisms of JARID2 regulation in OSCC as a predictive immunotherapeutic target.

However, the expression pattern of JARID2 and its correlation in OSCC patients is still unclear and requires further elucidation. In this study, we first analyzed the expression levels of JARID2 based on the data of OSCC samples from TCGA database, and observed a significant higher expression of JARID2 in OSCC samples compared to normal samples. We then proved that compared to normal oral epithelial cell lines and adjacent normal samples from OSCC patients, JARID2 expression is elevated in OSCC cell lines and cancer tissues. To explore more potential roles of JARID2 in OSCC, we conducted a series of bioinformatic analyses. Through co-expression analysis, we identified various genes with different functions that significantly correlated with JARID2, including NUP153, ITPR3, RNASEH2C, S100A13, etc. It was indicated that JARID2 was in an intricate gene network participating in the regulation of complicated molecular events in OSCC. By functional enrichment analyses based on GSEA & GSVA, JARID2 was showed to be involved with mitotic spindle, protein secretion, TGF-β, oxidative phosphorylation signaling pathways. In immune-related functional analysis, we found that the immune cells, which has a infiltration positively correlated with JARID2 expression, comprised not only immune cells that exerted anti-tumor functions (CD56bright natural killer cell, activated CD4 T cell, central memory CD4 and CD8 T cell, activated dendritic cell), but also immune cells that exerted immunosuppressive and tumor promoting functions (type 2 T helper cell, neutrophil, plasmacytoid dendritic cell). Similarly, the infiltrations of both anti-tumor immune cells (CD56dim natural killer cell) and tumor-promoting immune cells (monocyte, activated CD8 T cell) were observed to be negatively correlated with JARID2 expression. This demonstrated a highly complex tumor immune microenvironment associated with JARID2. Considering the diverse roles of immune cells in tumor progression, the immune-related functions of JARID2 still need more exploration. What’s more, heterogeneous gene mutation profiles and drug sensitivity for OSCC patients was revealed to be related to JARID2, which could contribute to targeted therapy for OSCC guided by JARID2.

## Conclusion

Exploring prognostic biomarkers for OSCC contributes to personalized treatment. Our research has initially observed upregulation of JARID2 in OSCC and has begun to investigate its association with enhanced cancer cell proliferation, invasion, and migration. Study of clinical samples from patients suggests that JARID2 may closely influence tumor staging and indicate a potential indicator of poor prognosis. Through bioinformatics analysis, we have attempted to predict the potential functions of this gene in OSCC as much as possible. However, the etiology or molecular function of JARID2 in OSCC has not been reported. Furthermore, these bioinformatics results require extensive experimental validation to be effective in future treatments. Future research should further study the complex interactions between different members within the chromatin structural domain of the PRC2 gene and explore the connection between JARID2 and the tumor immune microenvironment.

This study also has certain limitations. Firstly, as an initial investigation, we did not validate the expression and function of JARID2 at the protein level. We only conducted preliminary verification of its biological impact. Secondly, the carcinogenic mechanisms of JARID2 in OSCC have not been further analyzed in vivo, and there is a lack of exploration into the specific molecular and protein mechanisms involved in JARID2 regulation. However, we will continue to delve into this topic in subsequent studies and explore molecular research related to the immune microenvironment of OSCC.

## Data Availability

The original contributions presented in the study are included in the article.
